# Non-invasive EEG-based brain-computer interfaces in patients with disorders of consciousness

**DOI:** 10.1186/2054-9369-1-14

**Published:** 2014-07-14

**Authors:** Emilia Mikołajewska, Dariusz Mikołajewski

**Affiliations:** Rehabilitation Department, Military Clinical Hospital No. 10 and Polyclinic, Powstańców Warszawy 5, Bydgoszcz, 85-681 Poland; Disorders of Consciousness Research and Neurorehabilitation Unit, Neurocognitive Laboratory, Centre for Modern Interdisciplinary Technologies, Nicolaus Copernicus University, Toruń, Poland; Institute of Mechanics and Applied Computer Science, Kazimierz Wielki University, Bydgoszcz, Poland; Department of Applied Informatics, Department of Physics, Astronomy and Applied Informatics, Nicolaus Copernicus University, Toruń, Poland

**Keywords:** Neurological disorders, Disorders of consciousness, Brain-computer interfaces, EEG-based BCIs

## Abstract

Disorders of consciousness (DoCs) are chronic conditions resulting usually from severe neurological deficits. The limitations of the existing diagnosis systems and methodologies cause a need for additional tools for relevant patients with DoCs assessment, including brain-computer interfaces (BCIs). Recent progress in BCIs’ clinical applications may offer important breakthroughs in the diagnosis and therapy of patients with DoCs. Thus the clinical significance of BCI applications in the diagnosis of patients with DoCs is hard to overestimate. One of them may be brain-computer interfaces. The aim of this study is to evaluate possibility of non-invasive EEG-based brain-computer interfaces in diagnosis of patients with DOCs in post-acute and long-term care institutions.

## Introduction

Disorders of consciousness (DoCs) are chronic conditions resulting usually from severe neurological deficits. The most common are coma, vegetative state (VS)/unresponsive wakefulness syndrome, minimally conscious state (MCS, categorized recently into MCS + and MCS-), and locked-in syndrome (LIS) – constituting, according to some researchers, a continuum of consciousness. Patients with locked-in syndrome are considered as being fully conscious and therefore not part of patients with disorders of consciousness. LIS patients are fully conscious but unable to move and speak, so they can be diagnosed as VS patients. Damaged integration of system-level functional connectivity is perceived as one of the causes of DoCs. In VS and MCS the cause may be lack of external (perceptual) awareness, and internal (self-related) awareness related with the disruption of associated neural networks in selected brain areas, despite the preserved wakefulness networks of brainstem and basal forebrain [1–3]. But there is lack of one predominant paradigm in this area. This discrepancy results in severely decreased diagnostic accuracy and possible diagnostic mistakes. Moreover there is discussion even in the area of the number and names of DoCs [4–7]. Current diagnostic tools dedicated to patients with disorders of consciousness (e.g. unstandardized behavioral tests) seem to be insufficient, because such assessment relies mainly upon the subjective interpretation of observed behaviour.

The limitations of the existing diagnosis systems and methodologies should be detailed more. Observational diagnosis is based mainly by expertes on a list of items that the patient is unable to perform, thus is not objective (even 40% may need reclassification). Traditional methods and methodologias need improvement, then is neef for introducing more advanced, EEG-based or fMRI-based methods. EEG-based diagnosis is easy to set up, portable, widely available, practical for bedside testing, inexpensive, and provides very good temporal solution. Contrary fMRI (functional megnatic resonance imaging) is not portable and rather expensive, but has also non-invasive nature, and offers very good spatial resolution. But plastical changes caused by recovery process (e.g. in post-stroke patients) can provide activation patterns different from such patterns in healthy people, what makes challenge in analysis and interpretation. fNIRS (functional non-infrared spectroscopy) is more portable, low-noise, and artifact-sensitive than fMRI, and easier in everyday clinical use. But fNIRS is relatively new in BCIs applications, has limited depth of scanning (i.e. subcortical structures are hard to diagnose) and spatial resolution, and still needs for further research. Aforementioned limitations of the existing diagnosis systems and methodologies cause a need for additional tools for relevant patients with DoCs assessment [8], including brain-computer interfaces (BCIs). Clinical potential of current non-invasive EEG-based BCIs is not fully exploited, and need for further research on them should be emphasized. There is need for further technical development (signal gathering and processing, technical standarization, evaluation of commercial systems), standarized and wide accepted diagnostic battery. Thus evaluation of possibility of BCIs application in diagnosis of patients with DOCs in postacute and long-term care institutions still need for deeper research, clarification, and standarization, including clinical guidelines and procedures.

There are numerous healthcare problems, as far as ethical and social issues associated with DoCs, and there are additional issues associated with the therapy of patients with DoCs. Professionals working with patients with DoCs are at risk for developing burnout [8, 9]. Families and caregivers of patients with DoCs may additionally show various negative conditions, e.g. prolonged grief disorder (PGD) [10]. Increased assessment possibilities may significantly influence problem-focused coping strategies in the aforementioned group of people.

BCI is a technology that can utilize various neural imaging/recording modalities including fMRI, EEG, and even invasive recording of brain activities. Research on non-invasive BCIs have made important demonstrations in controlling communication aids and external devices. The objective of this article is to evaluate the possibility of non-invasive brain-computer interface's application in the diagnosis of patients with DoCs in post-acute and long-term care institutions. The content of this research is regarded as very relevant to technological and clinical perspectives. BCIs allow for real-time converting of the brain’s (bio)electrical activity into electrical signals for diagnosis, communication (using word processors or another dedicated software), and/or control (devices like neuroprostheses, wheelchairs, exoskeletons, etc., or even whole systems like smart home) purposes [11, 12]. (Bio)electrical activity of the central nervous system (CNS) is converted to a control signal without any peripheral (nervous) and/or muscular activity. This feature is perceived as very important for BCIs’ use in patients with DoCs.

## Material and methods

Review was limited to non-invasive EEG-based brain-computer interfaces applied in patients with disorders of consciousness. A review of publications indexed in three main data bases (Pubmed – U.S. National Library of Medicine, PEDro – Physiotherapy Evidence Database, Health Source: Nursing/Academic Edition) was conducted using the specified keywords (“brain-computer inteface”, “BCI”, “EEG”, “EEG-based”, “non-invasive”, “disorders of consciousness”, “DoC”, “coma”, “locked-in”, “vegetative state”, “P300”, and many more) as well as criteria of inclusion and exclusion (Table 1). The amount of BCI literature concerning both non-invasive and those using implanted-electrode interfaces is very large. Some BCI literature may not use the term DoC, but many papers mentioned fulfills specific conditions, including locked-in syndrome. Thus the search criteria may seem problematic, and the search keywords may seem problematic too.

**Table 1:** **The inclusion and exclusion criteria adopted in the review**

Inclusion criteria	Exclusion criteria
published after 2000	published before 2000
non-invasive EEG-based brain-computer interfaces	other kinds of non-invasive brain-computer interfaces, including fMRI-based, NIRS-based, etc.
English, other languages if English abstract available	English abstract not available
articles in reviewed journals	articles in unreviewed journals
recommended for medical professions	articles directed towards representatives of professions not connected with medical rehabilitation, e.g. sociologists etc.
editorial articles published in reviewed journals, letters to the editor, dissertations, conference abstracts, summaries of academic works, books or chapters in books	non-scientific articles

The synthesis of the representative publications and systematic quantitative analysis of previous studies and study results was conducted with the aim of presenting the scope as well as the importance of current academic research and concepts.

Our aim is to sufficiently explore BCI applications in the diagnosis of DoCs and their clinical significance. The methodology of the other works, possibilities, requirements, difficulties and results are presented below.

## Results

The authors conducted a search of three major databases using specified keywords and the aforementioned inclusion and exclusion criteria. The inclusion criteria was fulfilled by 29 publications for the years 2005–2014 (Table 2, Table 3).

**Table 2:** **Articles included for review**

Name of journal	Number of publications	Authors
Archives Italiennes de Biologie	1	Lehembre et al. 2012 [13]
Clinical Neurophysiology	5	Lulé et al. 2013 [14]
		Sellers 2013 [15]
		Murguialday et al. 2011 [16]
		Kübler & Birbaumer 2008 [17]
		Daltrozzo et al. 2007 [18]
Brain Injury	2	Chatelle et al. 2012 [19]
		Cavinato et al. 2009 [20]
Progress in Brain Research	3	Kübler & Neumann 2005 [21]
		Sorger et al. 2009 [22]
		Pfurtscheller 2006 [23]
Frontiers in Human Neuroscience	1	Risetti et al. 2013 [24]
Clinical EEG and Neuroscience	1	Lugo et al. 2014 [25]
Consciousness and Cognition	1	Tan et al. 2014 [26]
Annals of Neurology	2	Naci et al. 2012 [27]
		Steppacher et al. 2013 [32]
Conference Proceedings of the IEEE Engineering in Medicine and Biology Society	1	Eskandari & Erfanian 2008 [28]
Artificial Intelligence in Medicine	1	Pokorny et al. 2013 [29]
Current Opinion in Neurology	1	Kübler & Kotchoubey 2007 [30]
Neuroimage: Clinical	1	Chennu et al. 2013 [31]
PLoS One	1	Cavinato et al. 2012 [33]
Developmental Neurorehabilitation	1	Lancioni et al. 2011 [34]
Neuroimage	1	Chica et al. 2010 [35]
Journal of Cognitive Neuroscience	1	Van Gaal et al. [36]
Cognitive and Behavioral Neurology	1	Daltrozzo et al.2009 [37]
Neurocase	1	Schanakers et al. 2009 [38]
Neurology	1	Schnakers et al. 2008 [39]
International Journal of Rehabilitation Research	1	Uemura & Hoshiyama 2007 [40]
Neurocritical Care	1	Cruse et al. 2014 [41]
Total	29

**Table 3:** **Non-invasive EEG-based brain-computer interfaces in patients with disorders of consciousness – the review of reported studies**

Country – references, study group	Results	Remarks
Belgium – Lule et al. [14], N = 34	Four training trials and 10–12 questions “yes-no” showed functional communication in patients with locked-in syndrome and other patients with altered states of consciousness	BCI approaches have to be simplified to increase their sensitivity
UK - Kübler and Birbaumer [17], N = 35	Basic communication (yes/no) was restored in locked-in patients, bit not in any of the CLIS patients	BCIs application in CLIS patients still remains an open scientific problem
Italy – Cavinato et al. [20], N = 34	P300 was the only factor contributing to prediction of conscious recovery in patients in post-traumatic VS	
Italy - Risetti et al. [24], N = 11	High value of ERPs monitoring in DOC patients aiming at investigation of preserved conscious cognitive function	
Belgium, Lugo et al. [25], N = 6	P300 response to vibrotactile stimulation in patients with LIS.	
Germany, Steppacher et al. [28], N = 92	Significant relationship between N400 presence and subsequent recovery	
Austria, Pokorny et al. [30], N = 22	P300 accuracies were were insufficient for communication purposes in MCS patients	Further investigations are needed
UK, Chennu et al. [32], N = 29	Early, bottom-up P3a and the late, top-down P3b components in response to a pair of word stimuli may be regarded as signs of preserved attention	Further investigations are needed
Canada, Cruse et al. [41]	N20 and N35 somatosensory evoked potential (SSEP) show significant predictive value in patiens in coma	Research on etiology of the predictive power of these SSEP measures is needed

The representative literature was synthesized to indicate the scope and weight of current knowledge and experience. As discussed in the early work of Kübler & Neumann [20], the use of BCIs in severely paralyzed (locked-in) patients due to injury or disease is possible, but constitutes a huge challenge, and needs multidisciplinary research (comprising medical sciences, IT, biomedical engineering, cognitive sciences, psychology, etc.). According to Lehembre et al. [13], despite significant development of BCIs in the last twenty years there may be huge problems using them in patients with severe visual or auditory deficits, or severe lesions affecting their EEG signal. What is more, various etiologies of DoCs may additionally affect EEGs and/or Event Related Potentials (ERPs) in different ways [13]. Theses issues need further research. Even rather simple P300-based BCIs may be effective in behaviourally unresponsive patients [19]. There is no doubt in the potential for BCI's development, but the main limitations may be perceived as user training, simplicity, feedback, stimulation modality, sensitivity, and consistency [19]. As stated in the paper of Lulé et al. [14], only selected patients with MCS and LIS were able to use BCI based on the 4-choice auditory oddball EEG-BCI paradigm. Thus we should be aware that the proposed BCI's solution should be simplest (at the beginning, and then adoptable). On the other hand, only patients with completely locked-in syndrome (CLIS) are considered unable to use BCIs [15–17], but there still is an unusual case study by Schnakers et al. [38].

The diagnostic value of BCIs may be increased by the results that Mismatch Negativity (MMN) and P300 may be regarded as reliable predictors of awakening (conscious recovery) in low responsive patients [18, 24, 32–34, 37, 39], especially in patients in a vegetative state (VS) [21, 27] or LIS [25]. A similar role may be played by N400 [28], N200 [36], or P100 [35]. However, in untrained patients in an acute phase of LIS, novel hemodynamically-based BCIs (using fMRI, and functional near-infrared spectroscopy – fNIRS) may be predominant in the future [22].

The cortical activation model (CAM) by Pfurtscheller may improve the clinical significance of an event-related desynchronization (ERD) or event-related synchronization (ERS) in patients with DoCs [23].

Despite technical and clinical development BCIs for behaviourally unresponsive patients still present substantial challenges [27]. Preparation of the patient is important: mindfulness meditation training provided higher BCI accuracy compared to both the music training and no-treatment control groups [26, 29]. Novel paradigms offer opportunities to support the clinical assessment of DoCs, including MCS [30]. A whole hierarchical procedure in the area of the assessment of the DoCs patient’s cognitive abilities, consisting of passive stimulation, active instructions, volitional paradigms, and BCI operations was proposed by Kübler & Kotchoubey [31]. Further research provided deeper insight into the nature and capabilities of attention in patients with DoCs [32], exogenous orienting [35], and the relationship between the level of consciousness and cognitive control (e.g. if cognitive control processes can be initiated unconsciously) [36], and semantic processing [37]. Changes of P300 in elderly patients with dementia described by Uemura & Hoshiyama showed both novel possibilities of BCIs use and technical challenges [40].

## Discussion

Considering all the manuscript, analysis of the topic is limited to research on non-invasive EEG-based brain-computer interfaces. This article is regarded as preliminary. Authors conduct own research on BCI applications in patients with DoCs within international InteRDoCTor (International-Interdisciplinary Research for Disorders od Consciousness in Toruń) research team. Results of the research will be published elsewhere.

Analysis of published findings up to this point supports the hypothesis that further application of BCIs in patients with DoCs may have an important positive influence on diagnostic precision and its features (e.g. its inter-rater reliability), and, as a result, may positively influence outcomes of the therapy (including rehabilitation). The main challenges both for scientists, engineers, and clinicians are as follows:

- easy training (or no-training) tools for the most severe cases of BCIs,
- clinical procedures of BCIs installation or implantation, including indications and contraindications,
- clinical procedures and guidelines for non-invasive BCIs’ application for diagnostic purposes, including supplementary BCIs’ use with fMRI, PET, conventional EEG, etc.
- patient safety precautions, including possible threats and effects of long-term BCI use,
- ethical and legal problems, e.g. concerning the balance between human intent and its interpretation by BCIs’ software within the decision-making process,
- newest diagnostic tools and research methodologies according to the Evidence Based Medicine paradigm, including magnetoencephalography (MEG),
- whole families of scalable BCI devices and systems, depending on DoCs’ etiology, location(s) of lesion(s), patients’ clinical status, preserved cognitive functions, etc. (from simple yes/no communication to complex systems for communication and control).

Existing research has not sufficiently solved the aforementioned issues. The increased effectiveness of the therapy, the shortening of the hospitalization period, and significantly increasing the quality of life of patients with DoCs and their families/caregivers is worth every effort in the aforementioned area. The application of aforementioned occupations in a group of new medical services (telemedicine, telerehabilitation), and within an eclectic/mixed approach to intervention in neurorehabilitation seems to be obligatory.

## Conclusions

Recent progress in clinical applications of the non-invasive EEG-based BCI’s may offer important breakthroughs in the diagnosis and therapy of patients with DoCs, especially compared with traditional observational diagnosis. There is a need for further research concerning clinical application of non-invasive EEG-based BCIs’ for diagnosis, and, where possible, communication and control purposes. Implementing more sophisticated data analysis methods and neurofeedback training techniques may be necessary [22]. This approach still needs additional research. Moreover it is not accurate to say that current “traditional” diagnostic approaches to diagnosis in DoCs are not sufficient for managing DoC patients, and fMRI, fNIRS, MEG, etc. will be much better. It seems that a complex approach joining several diagnostic methods, techniques, and tools may dramatically increase the exactness of the diagnosis in patients with DoCs. Such hybrid solutions (incorporating e.g. non-invasive EEG-based BCI’s and fMRI) [42] are perceived important direction of further research within clinical application of BCIs in DoCs patients. Basic research on neuroimaging and electophysiology in patients with DOC may constitute a solid basis for further clinical research on identifying more advanced physiological and computational measures closely associated with level of consciousness [43–45].

## Authors’ information

Emilia Mikołajewska: http://www.emikolajewska.netstrefa.eu/.
